# Multiparametric Contrast-Free MRI Successfully Identifies Venous Thrombus Responsive to Lytic Therapy: From Mice to Humans

**DOI:** 10.1161/CIRCIMAGING.125.018175

**Published:** 2025-11-03

**Authors:** Justinas Silickas, Alberto Smith, Marcelo E. Andia, René M. Botnar, Bijan Modarai, Narayan Karunanithy, Ashish S. Patel, Stephen Black, Prakash Saha, Alkystis Phinikaridou

**Affiliations:** School of Cardiovascular and Metabolic Medicine & Sciences (J.S., A.S., B.M., A.S.P., S.B.), Kings College London, UK.; King’s British Heart Foundation Centre of Research Excellence (A.P.), Kings College London, UK.; School of Biomedical Engineering and Imaging Sciences, Research Department of Cardiovascular Imaging (R.M.B., N.K., P.S.), Kings College London, UK.; Radiology Department, School of Medicine, Pontificia Universidad Católica de Chile, Santiago (M.E.A.).; Millennium Institute for Intelligent Healthcare Engineering, Santiago, Chile (M.E.A., R.M.B.).; Escuela de Ingeniería (M.E.A.), Pontificia Universidad Católica de Chile, Santiago.; Institute for Biological and Medical Engineering (R.M.B), Pontificia Universidad Católica de Chile, Santiago.; Department of Vascular Surgery (B.M., A.S.P., S.B., P.S.), Guy’s and St. Thomas’ NHS Trust, London, UK.; Department of Interventional Radiology, (N.K.), Guy’s and St. Thomas’ NHS Trust, London, UK.

**Keywords:** fibrin, lung, magnetic resonance imaging, patient selection, venous thrombosis

## Abstract

**BACKGROUND:**

Randomized trials of venous thrombolysis to prevent postthrombotic syndrome have produced mixed results. A method to identify patients most likely to benefit from interventional treatment is needed. This study evaluated a contrast-free, magnetic resonance-based multisequence thrombus imaging (MSTI) technique to characterize deep venous thrombi and predict susceptibility to thrombolysis.

**METHODS:**

Venous thrombosis was induced in the inferior vena cava of BALB/C mice (n=56, male), which were imaged up to 28 days postsurgery and 24 hours after systemic thrombolysis (Actilyse, 10 mg/kg, IV). The prelysis MSTI protocol included 3-dimensional T1 mapping, 3-dimensional magnetization transfer, and 2-dimensional diffusion-weighted imaging. Thrombolysis was defined as successful if inferior vena cava blood flow increased by ≥50% compared with prelysis values. In a clinical cohort, 41 patients with acute iliofemoral deep venous thrombi underwent MSTI before catheter-directed thrombolysis. Imaging parameters were analyzed against postintervention outcomes.

**RESULTS:**

MSTI identified thrombi susceptible to thrombolysis in both mice and humans. In mice, lysed thrombi showed lower T1 (723 [667–782] versus 874 [799–1000] ms; *P*<0.001) and higher apparent diffusion coefficient values (1.02 [0.96–1.14] versus 0.78 [0.62–0.88]×10^-^³ mm²/s; *P*<0.001) than nonlysable thrombi, with no difference in magnetization transfer. In patients, lysed thrombi demonstrated lower T1 (606 [543–656] versus 765 [630–909] ms; *P*<0.001), lower apparent diffusion coefficient (0.67 [0.5–1.1] versus 1.23 [0.69–1.74]×10^-^³ mm²/s; *P*=0.001), and similar magnetization transfer rates. Combining MSTI parameters optimized prediction, achieving 88% sensitivity and 97% specificity in mice, and 86% sensitivity and 91% specificity in humans.

**CONCLUSIONS:**

MSTI enables noninvasive, contrast-free characterization of thrombus composition and predicts thrombolytic susceptibility. This technique has the potential to guide patient selection for invasive therapies and should be incorporated into future trials of venous thrombosis treatment.

**CLINICAL PERSPECTIVE**

The composition of venous thrombus is an important factor when considering interventional treatment. Acute, fibrin-rich thrombi are typically amenable to lysis, whereas older thrombi are more collagenous and therefore, resistant to plasmin-mediated degradation. However, current diagnostic tools cannot characterize thrombus composition and do not reliably identify venous thrombi suitable for thrombolytic treatment, limiting precise patient selection. Clinical decision-making, therefore, remains based on indirect indicators, such as symptom duration or anatomic burden, which do not accurately reflect underlying biology. A safe, fast, and noninvasive method that can objectively and accurately characterize the molecular composition of venous thrombi would therefore be desirable in clinical practice. Here, we developed a clinically applicable multiparametric magnetic resonance imaging scan, called multisequence thrombus imaging, that can characterize the composition of deep vein thrombus in vivo. We demonstrate that multisequence thrombus imaging enables noninvasive, contrast-free characterization of thrombus composition and accurately predicts thrombolytic susceptibility, offering a potentially transformative tool for treatment planning. Multisequence thrombus imaging could be used to accurately differentiate between acute and older thrombi in patients. This technique has clinical potential to precisely identify patients suitable for interventional treatments aimed at restoring venous patency.

**See Editorial by Dai and Zheng**

Deep vein thrombosis (DVT) is a common and potentially life-threatening condition, affecting ≈1 in 1000 people annually in the developed world.^1,2^ Although DVT can occur in patients of any age, it typically presents as a hot, painful, and swollen limb. In rare cases, it may progress to limb-threatening ischemia or result in life-threatening complications such as pulmonary embolism that can lead to sudden death.^3,4^ The initial management of DVT involves anticoagulation to prevent thrombus propagation and reduce the risk of embolization to the lungs,^5^ but this does not accelerate natural thrombus resolution,^6^ which takes place over a variable amount of time.

Despite treatment, approximately half of patients experience incomplete thrombus resolution, leading to chronic symptoms of postthrombotic syndrome (PTS).^7,8^ PTS is characterized by persistent pain, swelling, venous claudication, and, in severe cases, leg ulceration. This condition not only significantly reduces quality of life^9^ but also places a considerable financial burden on health care systems and patients due to the need for long-term care and repeated hospitalizations.^7,9^

To reduce the incidence of PTS, interventional treatments for DVT have been proposed. Recent years have seen an increase in the use of endovascular techniques, including catheter-directed thrombolysis (CDT), mechanical thrombolytic systems, thrombus aspiration, and thrombus retrieval devices. Combined with dedicated venous stents, these interventions aim to restore venous patency, promote early functional recovery, and reduce the long-term burden of PTS. However, patient selection is crucial, and invasive treatments, such as CDT, are thought to be most effective in fresh, fibrin-rich thrombi.^10^

Natural thrombus resolution is an intricate process that involves the replacement of an initial red blood cell-rich clot by fibrin, followed by the deposition of a collagenous extracellular matrix.^11^ Current clinical decision-making is not based on the underlying biology of the thrombus; however, it relies instead on indirect indicators or thrombus organization, such as symptom duration. But the timing of this organizational process varies between individuals, and since only fibrin-rich thrombi are typically amenable to lysis, the composition of a thrombus is an important factor when considering interventional treatment.^12,13^ A method that can objectively and accurately characterize the molecular composition of venous thrombi would therefore be desirable in clinical practice.

Although duplex ultrasound remains the first-line diagnostic tool for DVT, it cannot characterize thrombus composition. This has led to the development of a number of imaging techniques designed to better characterize human venous thrombi. These include strain elastography imaging,^14^ MR direct thrombus imaging,^15^ MR venography using both the flow-refocused fresh-blood imaging and the swap phase-encode arterial double-subtraction elimination.^16^ However, although these techniques provide age proxies, they lack quantitative molecular insight. Previous studies have demonstrated the potential of magnetic resonance imaging (MRI), both with and without contrast agents, to assess the structure of venous thrombi in experimental models.^11,17^ Building on this work, we have developed and optimized an MRI multisequence thrombus imaging (MSTI) technique that characterizes thrombus composition without the need for injectable contrast agents. In this study, we evaluate the efficacy of MSTI in predicting thrombus lysability in both experimental models and patients with DVT, to determine its potential for guiding thrombolytic therapy.

## Methods

### Data Availability

Data is not publicly available but may be made available on reasonable request to the corresponding author.

### Animal Model

Venous thrombosis was reproducibly induced in the inferior vena cava (IVC) of 12-week-old BALB/C mice in a surgical procedure that involves stenosis and endothelial disturbance as previously described.^11,17,18^ Details of the animal protocol are described in the Supplemental Methods; Figure S1A. All procedures were performed in accordance with the Animal (Scientific Procedures) Act 1986, under project license PP8419036.

### Patients With DVT

Ethical approval was obtained to investigate the role of novel MRI sequences to characterize DVT in patients (REC 13/LO/1472), and patients diagnosed with an iliofemoral DVT were included in this study. Diagnosis was made using a combination of patient history, clinical examination, D-dimer levels, ultrasonography, and cross-sectional imaging with either a 1.5T magnetic resonance venogram or computed tomography with contrast agent (Figure S1B). We recruited 50 patients with a diagnosis of an acute iliofemoral venous thrombosis, of whom 41 patients underwent MSTI before CDT and were included in the postinterventional analysis. The reasons for exclusion included declining interventional treatment following counseling by the clinical team (n=4), inability to tolerate MRI (n=2), and movement artifact limiting the ability to analyze the data fully (n=3). The patient demographics and procedural factors related to their intervention are shown in the Table.

**Table.**
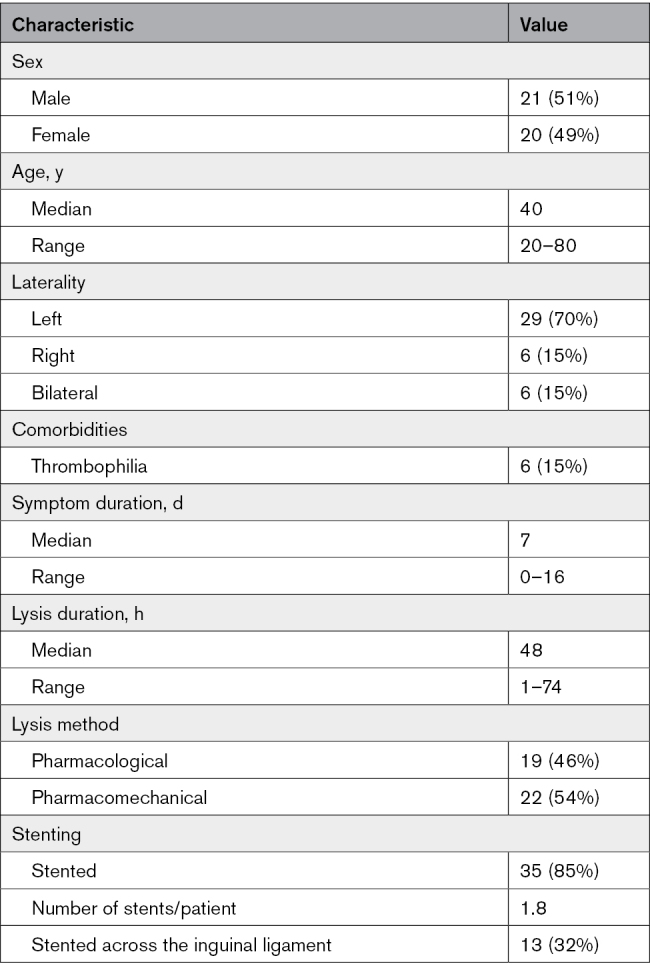
Patient Characteristics

### Magnetic Resonance Imaging

#### Mouse Imaging

MRI was performed using a 3T MR scanner (Philips Achieva, Philips Healthcare, Best, the Netherlands) equipped with a clinical gradient system (30 mT/m, 200 mT/[m·ms]) and a single-loop surface coil (diameter=47 mm) as we have previously described.^11,17,18^ Mice were imaged longitudinally at days 2, 4, 7, 10, 14, 21, and 28 (n=8 per time point) after thrombus induction. Following a 3-dimensional (3D) gradient echo scout scan, 2-dimensional (2D) time-of-flight angiography and venography images were acquired to identify the abdominal aorta and IVC, which were used as fixed anatomic landmarks. The filling defect attributable to the presence of thrombus in the IVC was used to identify the location and extent of the thrombus. Subsequently, the MSTI protocol to characterize thrombus composition was applied and included 3D T1-mapping, 3D magnetization transfer (MT), and 2D diffusion weighted scans.

#### Human Imaging

MRI was performed using the same 3-Tesla MRI scanner as for moue imaging, but using a 32-channel cardiac coil. Patients were imaged in the supine position, and the total acquisition time was 35 minutes. Following a 3D gradient echo scout scan, MR venography images were acquired to visualize the venous system in the segment of thrombus obstruction using 3D balanced steady-state-free precession and 2D time-of-flight scans. T1 mapping was performed using a 2D Modified Look-Locker Inversion Recovery sequence with an acquisition scheme of 3 to 5.^19,20^ T1-weighted spoiled 3D gradient echo images were acquired (1) without and (2) with an on-resonance MT prepulse.^17^ Finally, 2D diffusion weighted images were acquired with diffusion gradients applied parallel and perpendicular to the external magnetic field. Specific details of all imaging procedures are given in the Supplemental Methods, with comparison of humans and mouse protocols shown in Table S1. Before the clinical study, the MSTI protocol was validated in 10 healthy volunteers to optimize coil positioning, scan duration, and anatomic coverage. This allowed adjustment of acquisition parameters to ensure image quality and reproducibility across targeted venous segments.

### Thrombolysis

#### Murine Thrombolysis

Mice were imaged between days 2 and 28 following thrombus induction (Figure S1A). Immediately following each MRI scan, tPA (tissue-type plasminogen activator solution, Actilyse) was infused by a tail-vein injection over 5 minutes (10 mg/kg). Phase-contrast images were used to estimate blood flow in the IVC before and 24 hours after thrombolytic therapy. Thrombolysis was considered successful if there was an increase of at least 50% in IVC blood flow compared with prelysis values.

#### CDT in Patients

Catheter-directed thrombolysis was performed using a standardized protocol. In most instances, the ipsilateral popliteal vein was punctured under ultrasound guidance, and the lesion was crossed using fluoroscopy. Standard venography was performed to localize the thrombus and position a Cragg-McNamara infusion catheter to deliver lytic therapy directly into the thrombus. tPA was used at a maximum dose of 1 mg diluted in normal saline, administered at 10 mL/h. Heparin was concurrently administered via the sheath. A check venogram was performed at 24 hours following the start of lysis to assess the degree of thrombus resolution. If >50% stenosis of the vessel diameter remained as a result of residual thrombus, lysis was continued for another 24 to 48 hours with daily check venograms. On completion of the lysis protocol, the vessels were assessed using contrast venography and intravascular ultrasound. Successful lysis was defined as a completely cleared venous segment with <50% residual vessel stenosis. Failure of lysis was defined as stenosis of >50% of vessel lumen diameter, residual thrombosis visible on venography or intravascular ultrasound, venoplasty with visible balloon wasting, and the need for venous stenting. Final check venography was performed to demonstrate rapid clearance of the contrast agent and disappearance of drainage through collateral vessels. All patients received an initial 24-hour infusion of CDT. This consistent therapeutic window formed the basis for MSTI analysis. Any further interventions (eg, thrombectomy or stenting) were not included in predictive modeling but are reported for clinical context in the Table.

### MR Image Analysis (Mouse and Human)

MRI images covering the region of venous obstruction, as seen on MR venography, were analyzed on a slice-by-slice basis to derive the T1 relaxation time (s), percentage of magnetization transfer rate (% MTR), and apparent diffusion coefficient (ADC, mm^2^/s) values to describe thrombus composition. The time-of-flight images were used to identify the IVC and the abdominal aorta, which were used as fixed anatomic landmarks. The filling defect attributable to the thrombus in the IVC was used as a measure of thrombus volume. Regions of interest (ROI) encompassing the thrombus were segmented using OsiriX (OsiriX Foundation, Geneva, Switzerland) on the MR venography images and subsequently copied to the multi-sequence images. T1 maps were automatically generated using the scanner software after acquisition using a 3-parameter fit model. The ROIs defined by venography were copied onto the T1 maps to calculate the mean T1 relaxation time (s). The same ROIs were then propagated onto the images acquired with and without MT to calculate the % MTR based on the following formula: %MTR=(M_0_−M_s_)/M_0_×100, where Mo is the mean signal intensity without the MT prepulse, and Ms is the mean signal intensity with the MT prepulse. MTR maps were generated based on the same formula using an Osirix plug-in for visualization of macromolecule (eg, protein) rich areas within the thrombus. Finally, the same ROIs were used to calculate the ADC using the following equation: ADC=ln (S_0_−S_i_)/(b_i_b_0_; mm^2^/s), where S_i_ is the signal intensity of the area of interest obtained with a b value, b_i_. ADC maps were generated based on the same formula using an Osirix plug-in for visualization of the porosity/diffusivity of protons within the thrombus microenvironment. Thrombus segments with ADC values exceeding 2.0×10^−^³ mm²/s were excluded from analysis due to the likelihood of artifact or flow-related error. This threshold was selected based on prior experience and to reflect diffusivity values above the physiological range.

### Analysis of Patient With DVT Data Following CDT

Segments of the vasculature were anatomically divided for analysis into the common iliac vein, external iliac vein, and common femoral vein. Each segment was graded at 24 hours following the start of CDT as success or failure based on its appearance at check venography. If there was >50% restoration of luminal flow, lysis was considered successful. Intravascular ultrasound was used in all patients to confirm luminal patency on procedure completion. Image segmentation in patients is illustrated in Figure S2; Supplemental Video.

Interobserver reproducibility was assessed in the first 12 consecutive patients, representing approximately one-quarter of the intended recruitment target of 50. This subset was considered sufficient to validate segmentation and parameter extraction while keeping dual analysis feasible. Once substantial agreement was demonstrated, the remainder of the cohort was analyzed by a single observer to ensure consistency and efficiency.

### Statistical Analysis

Statistical analysis was performed using SPSS Statistics (IBM Corp, Armonk, NY) and GraphPad Prism (GraphPad Software, San Diego, CA). Data was tested using the Shapiro-Wilk test, showing non-normal distribution; therefore, nonparametric statistical tests were used. A Mann-Whitney *U* test was used to compare MSTI values of lysable and nonlysable thrombi. Correlation between 2 observers was calculated using the Pearson correlation test, interobserver agreement, using the Bland-Altman method for numerical values, and Cohen κ for categorical variables. Receiver-operating characteristic curve analysis was performed to assess the diagnostic ability of individual sequences and select lysability thresholds. Data are presented as medians and interquartile ranges. *P*<0*.05* was considered indicative of a significant difference.

Univariate binary logistic regression was performed using T1, MTR, and ADC as the 3 significant values, followed by multivariate binary logistic regression analysis that identified T1 and ADC as the 2 significant variables. Since we used a multivariable logistic regression model, β₀ represents the intercept, β₁ is the coefficient for T1, and β₂ is the coefficient for ADC, each indicating the change in the log-odds of lysis per unit change in the predictor. These coefficients (β₀=5.37, β₁=–0.006, β₂=–0.448) were then used to construct the lysis predictability model (Equation 1).


$$\begin{array}{l} P=\frac{e^{\left(\beta_{0}+\beta_{1}\cdot T_{1}+\beta_{2}\cdot ADC\right)}}{1+e^{\left(\beta_{0}+\beta_{1}\cdot T_{1}+\beta_{2}\cdot ADC\right)}} \end{array}$$ (1)

To make our predictability model more clinically useful, we reconstructed thrombus volume in 3D and created a lysis probability heat map illustrating how likely each slice of the thrombus is to lyse (with red indicating completely unlysable thrombi and green for fully lysable thrombi). The probability of lysis defined by MSTI was then validated based on the postoperative thrombolysis outcome. Segments of the thrombus identified as lysable by MRI showed a patent vein on postoperative venography. Conversely, segments of the thrombus with a low probability of lysis, as defined by MSTI, required stenting to restore blood flow.

## Results

### MSTI Differentiates Between Experimental Venous Thrombi That Lyse From Those That Do Not Lyse

**Figure 1.**
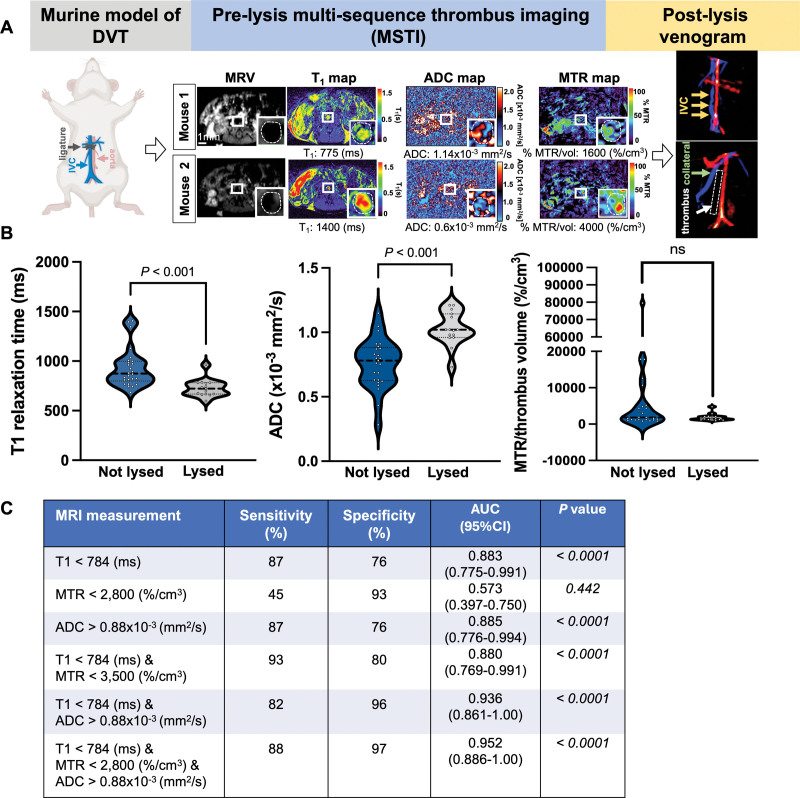
**Application of multisequence thrombus imaging (MSTI) in the murine model of deep vein thrombosis (DVT). A**, Schematic of the murine model of DVT followed by prelysis MSTI including MR venography (MRV), T_1_ mapping, magnetization transfer rate maps (MTR maps), and apparent diffusion coefficient (ADC) maps. The thrombus (white box) is magnified and segmented (insert and dashed lines to indicate regions of interest [ROIs]) to calculate the average T_1_ relaxation time, MTR normalized to thrombus volume, and ADC values. Postlysis MRV (24 hrs) showing successful lysis in mouse 1 and failed lysis in mouse 2. **B**, Violin plots of quantitative MSTI data show the differences between lysed and nonlysed thrombi. **C**, Table showing the performance of MSTI in detecting thrombi that lyse successfully. Receiver operating characteristic (ROC) analysis with area under the curve (AUC) is presented for each MSTI parameter and its combination.

Based on our previous work,^11,17,18^ an MR venogram, flow-encoded venography, T1 mapping, MT, and diffusion-weighted sequences were applied to mice with venous thrombi of varying ages before and 24 hours after tail vein administration of a lytic agent, tPA (Figure 1A). Quantitative analysis of MR sequences revealed significant differences in ADC (*P*<0.001) and T1-relaxation times (*P*<0.001), but no difference in MTR between lysed and nonlysed segments of thrombi (Figure 1B). Thrombi that lysed had significantly lower T1 (T1=723 [667–782] versus 874 [799–1000] ms; *P*<0.001) and higher ADC values (1.02 [0.96–1.14] versus 0.78 [0.62–0.88] 10^−3^mm^2^/s; *P*<0.001) compared with thrombi that did not lyse. Sequences were next combined to improve the sensitivity and specificity of MRI in differentiating thrombi that lyse from those that do not. Receiver-operating characteristic curves were used to identify the cutoff values that best detect thrombi susceptible to lysis. When all sequences were combined, a T1-relaxation time <784 ms, MTR <2800%/cm^3^, and ADC >0.88 mm^2^/s gave a sensitivity of 88% and specificity of 97% in differentiating lysable from nonlysable segments of a thrombus (Figure 1C).

### Translating MSTI Into Patients With DVT Undergoing CDT

Having identified candidate sequences in the experimental model of DVT, we adjusted the MSTI protocol in 10 healthy volunteers to facilitate its clinical use. To achieve that, the imaging volume was increased to cover the anatomic region extending from the IVC to the common femoral vein, a multitransmit coil that enabled accelerated image acquisition, adjusted the image resolution to achieve clinical evaluation of the thrombus within 35 minutes, and optimized the patient positioning on the table and the location of the coil. All patients included in the analysis underwent MSTI imaging before CDT, and these scans were used to segment the thrombus and extract imaging parameters on a slice-by-slice basis. The median age at intervention was 40 years (range, 20–80 years), and the median reported symptom duration at presentation was 7 days (range, 0–16 days). The median lysis duration was 48 hours (range, 1–74 hours), with an average of 59 mg of tPA used per patient. Placement of a venous stent following completion of lysis was performed in 35 patients (85%).

**Figure 2.**
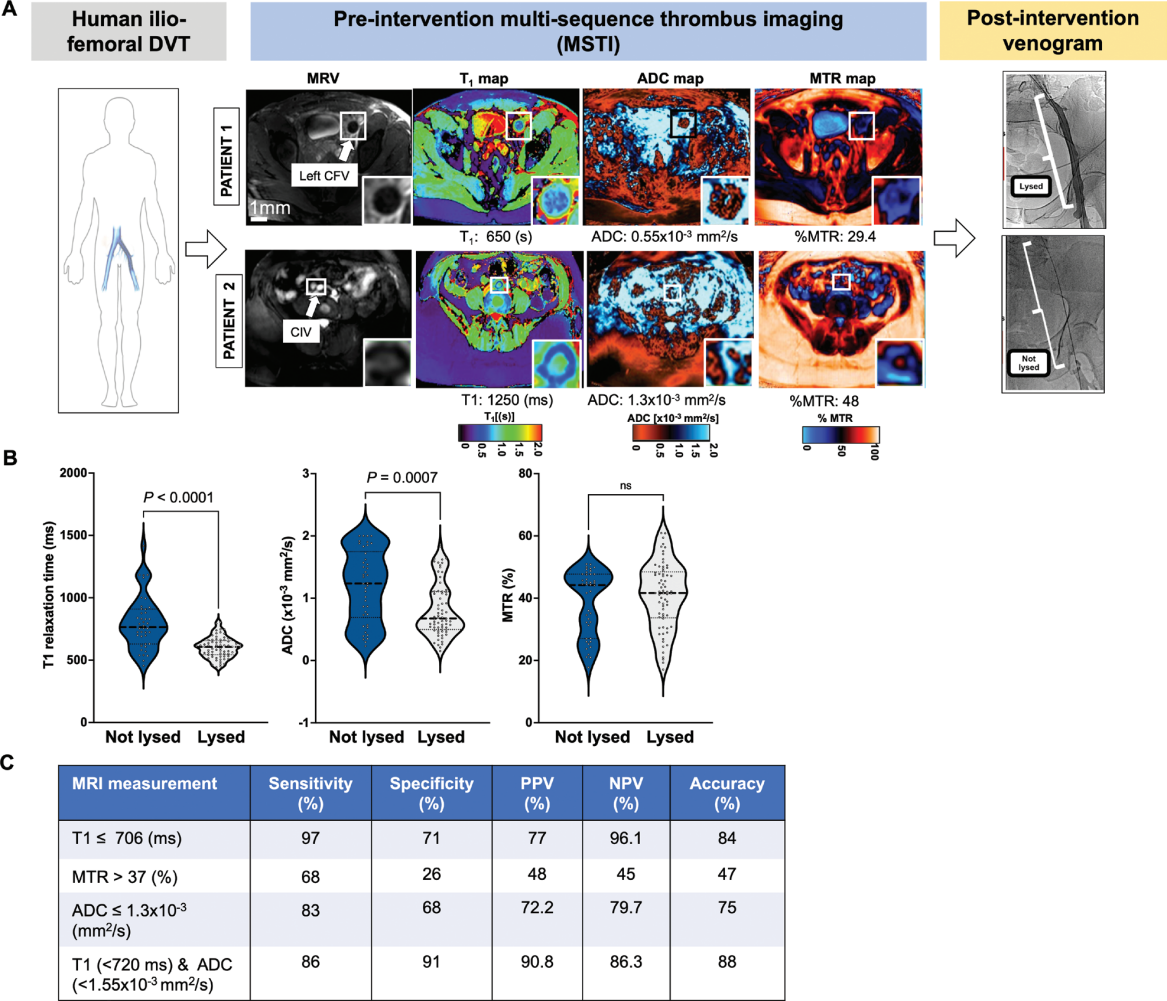
**Application of multisequence thrombus imaging (MSTI) in patients with deep vein thrombosis (DVT). A**, Schematic showing the anatomic region of the prelysis MSTI scan, followed by prelysis MSTI images including MR venography (MRV), T_1_ mapping, magnetization transfer rate maps (MTR maps), and apparent diffusion coefficient (ADC) maps. The thrombus (shown also in the inserts and in dashed lines) was segmented to calculate the average T_1_ relaxation time, MTR normalized to thrombus volume, and ADC values. Postlysis MRV shows successful lysis in patient 1 and unsuccessfully lysis in patient 2. **B**, Violin plots of quantitative MSTI data show the differences between lysed and nonlysed thrombi. **C**, Table showing the performance of MSTI in detecting thrombi that lyse successfully. CFV indicates common femoral vein; CIV, common iliac vein; NPV, negative predictive value; and PPV, positive predictive value.

Representative preoperative images of thrombi acquired with the MSTI protocol and postintervention venograms are illustrated in Figure 2A. Segmentation of the thrombus on the preoperative MSTI scans revealed significant differences in all 3 types of MRI images between lysed and nonlysed thrombi (Figure 2A). There was a significant difference between lysed and nonlysed thrombus for T1 relaxation times (606.1 [543–656] versus 765 [630–909] ms; *P*<0.0001) and ADC values (0.67 [0.5–1.1] versus 1.23 [0.69–1.74]×10^−3^mm^2^/s; *P*=0.0007) but similar MT rates (41.64 [33.67–48.44] versus 44.17 [27–47.68]; *P*=0.45). Similar to our data in the mouse model of DVT, combinations of these sequences showed better discriminatory power than just T1 alone, although in patients with DVT, unlike the mouse, lower ADC values were associated with lysis. Receiver-operating characteristic curve analysis of these data demonstrated that the best discrimination of the susceptibility to lysis was obtained when the T1 relaxation time and ADC were combined with an 86% sensitivity and 91% specificity (Figure 2C). Receiver-operating characteristic analysis is presented in Table S2.

Inter-observer agreement was assessed in the first 12 patients. There was strong inter-observer correlation for all 3 MRI sequences (T1 *r*=0.92, *P*<0.001; MTR *r*=0.88, *P*<0.001; and ADC *r*=0.94, *P*<0.001; Figure S3A). Bland-Altman plots demonstrated that there was no significant bias in any of the MRI parameters measured (Figure S3B). Interobserver agreement in assessment of venography (thrombus lysed versus nonlysed) using Cohen κ was 0.66 (95% CI, 0.58–0.74), showing substantial agreement between 2 independent assessors.

### Predicting the Lysability of a Thrombus Based on MSTI

**Figure 3.**
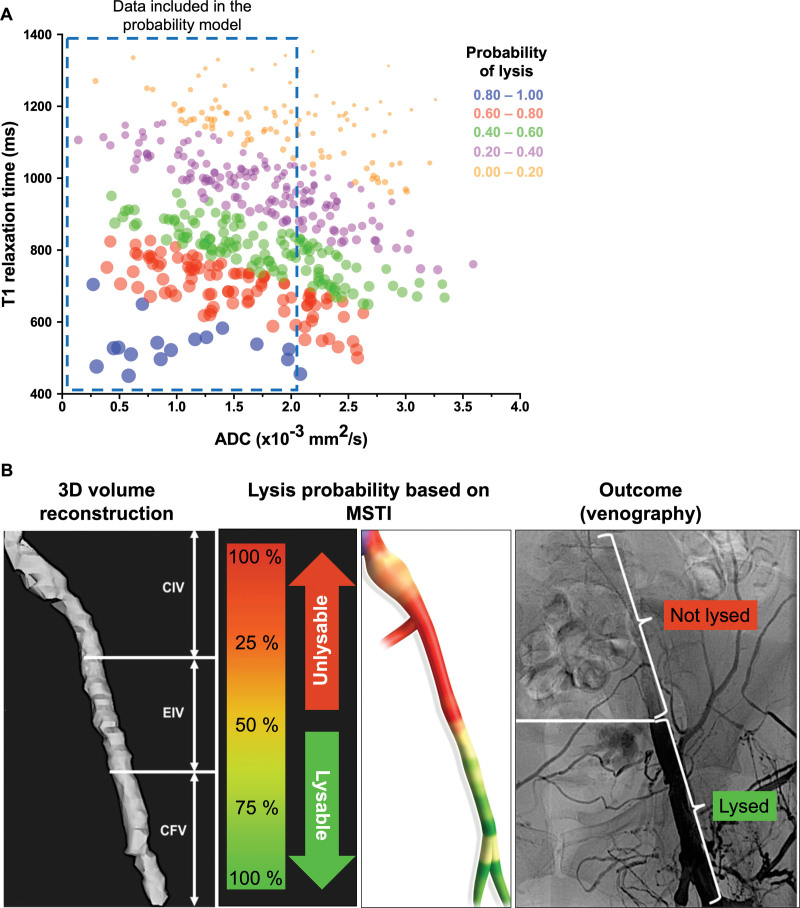
**Predicting the lysability of a thrombus based on multisequence thrombus imaging (MSTI) performed in patients with deep vein thrombosis (DVT). A**, Probability of lysis predicted based on coefficients from the logistic regression using the MSTI data (T1 relaxation time and apparent diffusion coefficient [ADC]). Each data point represents a single axial thrombus segment from the 41-patient cohort. Segmental MSTI values were input into the multivariable model to generate probability scores, with color coding as shown to indicate lysis probability. The blue box indicates the data included in the predictability model. **B**, Three-dimensional (3D) reconstruction of thrombus volume using magnetic resonance imaging (MRI) data. A corresponding heat map demonstrates the lysis probability, calculated using MSTI data, on a slice-by-slice basis. Red color indicates unlysable thrombi and green indicates lysable thrombi. This case is shown as a conceptual example of how MSTI might be visualized clinically. Postoperative X-ray venography shows the outcome of thrombolysis (black=successful lysis with blood flow). CFV indicates common femoral vein; CIV, common iliac vein; and EIV, external iliac vein.

Univariate binary logistic regression was performed using T1 (odds ratio [OR], 0.988 [0.984–0.993]; *P*<0.001), MTR (OR, 1.020 [0.984–1.059]; *P*=0.45), and ADC (OR, 0.219 [0.093–0.4833]; *P*<0.001), followed by multivariate binary logistic analysis that identified T1 (OR, 0.989 [0.984–0.993]), and ADC (OR, 0.398 [0.152–1.000], as the 2 significant variables (area under the curve, 0.847, [0.764–0.930]; *P*<0.0001). These were then used to construct a lysis predictability model, using the coefficients from the logistic regression described above to calculate the predicted probability of lysis for that slice through the thrombus. Thrombi having a low T1 relaxation time and low ADC values had the highest probability of lysis (blue dots). This probability decreases with increasing T1 and ADC values. Figure 3A includes all thrombus segments from the entire patient cohort (n=41). Each dot represents 1 segment analyzed using MSTI parameters on a slice-by-slice level.

To make our predictability model easier to interpret, we reconstructed thrombus volume in 3D and created a lysis probability heat map illustrating how likely each slice of the thrombus is to lyse (with red indicating completely unlysable thrombi and green for fully lysable thrombi). This conceptual representation of how MSTI-derived probability maps could be applied in the future for individualized patient care is shown in Figure 3B. The probability of lysis defined by MSTI was then validated based on the postoperative thrombolysis outcome. Segments of the thrombus identified as lysable by MRI showed a patent vein on postoperative venography. Conversely, segments of the thrombus with a low probability of lysis, as defined by MSTI, required stenting to restore blood flow.

## Discussion

In this study, we developed and validated a clinically translatable, noncontrast agent MRI-based imaging method, MSTI, that characterizes venous thrombus composition and predicts susceptibility to thrombolytic therapy. Our results show that MSTI reliably differentiates between lysable and nonlysable thrombi with high sensitivity and specificity, providing an objective, noninvasive approach for selecting patients who may benefit from CDT. Given the challenges in DVT management, particularly the variable outcomes with thrombolysis, our findings have significant clinical implications.

CDT has been proposed as 1 strategy to enhance thrombus clearance and potentially reduce the incidence of PTS, a long-term complication of DVT that can lead to chronic pain, swelling, and venous dysfunction.^21^ However, the efficacy of CDT remains controversial, as demonstrated by the mixed results from randomized controlled trials, such as the ATTRACT trial.^10^ One possible reason for the inconsistent outcomes may be the reliance on symptom duration as a surrogate for thrombus age and lytic potential. Symptom onset is often subjective and biologically imprecise. Recent translational work by Li et al^12^ demonstrated that thrombolysis was most effective when administered within a 4 to 8 day window after symptom onset, but this window reflects population-level timing and cannot account for individual thrombus heterogeneity. MSTI addresses this limitation by offering a direct and patient-specific method to characterize thrombus biology, moving beyond arbitrary time cutoffs. Our study suggests that thrombus composition, rather than age alone, is a more reliable predictor of successful thrombolysis, as evidenced by the superior accuracy of MSTI in identifying lysable thrombi compared with clinical history alone. This finding aligns with early foundational work, which showed that aged thrombi exhibited reduced responsiveness to tPA despite dose escalation,^13^ highlighting the role of thrombus organization as the limiting factor.

The findings of this study are consistent with our previous work and the work of others,^22–25^ which demonstrated that fibrin-dense thrombi are more responsive to thrombolytic therapy, while highly organized thrombi with significant collagen deposition are less amenable to lysis. This highlights the critical role of thrombus structure in determining treatment success. MSTI advances this concept by providing a contrast-free, quantitative MRI-based assessment of these compositional features. As red blood cells degrade and macrophages accumulate within thrombi, paramagnetic iron breakdown products accumulate, accelerating spin-lattice relaxation and shortening T1 values. This molecular signal allows MSTI to identify thrombi enriched in fibrin and iron, features associated with better thrombolytic outcomes.

Our earlier studies also showed that low T1 relaxation times are associated with high fibrin content, while lower ADC values indicate increased thrombus porosity, both factors crucial for thrombolytic response.^17^ In this study, we extended these findings to a clinical setting, demonstrating that MSTI can reliably predict thrombus response to CDT. Overall, T1 mapping was the most consistent and clinically feasible predictor of thrombolytic response. The addition of ADC provided a modest improvement in specificity and was more informative in the murine model than in patients. Although these findings suggest that T1 alone may be sufficient for many clinical applications, we felt it important to report ADC performance in this initial translational work, as future refinements in diffusion imaging could enhance its utility. Interestingly, in contrast to our murine models, where lower ADC values typically reflect higher thrombus permeability, these lower ADC values were also associated with successful lysis in patients. This suggests that thrombus diffusivity may reflect more complex microstructural differences in human DVT, including iron-mediated susceptibility effects and extracellular matrix constraints, which require further study.

Multiple additional factors, including red blood cell^24^ content, fibrin structure, extracellular DNA nets, and local blood flow dynamics, may influence thrombus susceptibility to lysis.^26–28^ Unlike traditional imaging methods, such as ultrasound or contrast-enhanced CT, which focus primarily on thrombus size and location, MSTI has the potential to guide patient selection more precisely and eliminates the need for further cross-sectional imaging when planning an intervention, by providing a detailed, noninvasive evaluation of thrombus composition, in addition to essential information on thrombus size and location. These advantages are particularly valuable in ambiguous cases where clinical history is unreliable or thrombus organization is heterogeneous.

Beyond its potential application in thrombolytic therapy, MSTI may also play a role in guiding the use of emerging thrombus removal strategies. These include purely mechanical methods, such as thrombectomy devices, and pharmacomechanical therapies, which combine mechanical clot disruption with local administration of thrombolytic agents. Both strategies have shown promise in improving thrombus clearance, particularly in cases where thrombolysis alone may be insufficient due to the advanced organization of the thrombus or resistance to lytic agents.

We propose that MSTI could help identify patients who are more likely to benefit from 1 treatment approach over another. For example, highly organized thrombi with extensive collagen deposition may respond better to mechanical thrombectomy, whereas fibrin-rich thrombi may be more amenable to pharmacomechanical approaches or thrombolysis alone. Further studies are needed to explore how MSTI could guide the choice of intervention and to assess its role in the context of these emerging therapies.

The clinical translatability of MSTI is a key strength of this study. Unlike imaging techniques that require exogenous contrast agents, MSTI uses MRI sequences that are widely available on clinical scanners, facilitating rapid integration into routine practice. We optimized the protocol for clinical feasibility, ensuring that it can be applied in the diagnostic workflow without significant time delays. Furthermore, MSTI’s noninvasive nature allows for longitudinal monitoring of thrombus resolution, which could be crucial in assessing treatment efficacy and guiding ongoing management decisions.

### Study Limitations and Future Directions

While MSTI represents a significant advance in thrombus imaging, several limitations should be acknowledged. (1) MSTI provides a surrogate measure of thrombus structure and does not directly visualize molecular components such as fibrin or collagen. Future studies incorporating molecular imaging techniques, such as positron-emission tomography-MRI with fibrin-targeted tracers that also showed a direct relationship between T1 mapping and fibrin content in thrombus^29,30^ could provide a more comprehensive understanding of thrombus composition but will require the use of a contrast agent. (2) The widespread adoption of MRI-based approaches like MSTI may be limited by the availability and cost of MRI scanners. However, emerging MRI technologies, including faster acquisition protocols and deep-learning-based image reconstruction, may help mitigate these challenges and improve accessibility. (3) Planning and segmentation of 2D acquisitions require training and experience, and careful standardization will be important for reliable implementation across centers. (4) The time required for postprocessing and image analysis, which was carried out manually in this study, remains significant. To address this, we are developing a deep-learning segmentation pipeline to automate ROI placement, feature extraction, and prediction heatmap generation. This could make MSTI practical for rapid interpretation in high-volume centers. Moreover, because this study used a 32-channel cardiac coil on a single scanner platform at a single institution, further work will be needed to assess how MSTI can be implemented across centers using different coils, manufacturers, and sequence configurations. Prospective multicenter validation will also be essential to determine generalizability, clinical utility, and cost-effectiveness.

Looking ahead, further research should focus on refining the MSTI protocol, including the application of motion-compensated diffusion gradients in any future work and integrating advanced MRI techniques, such as magnetic resonance fingerprinting,^31^ which could offer additional quantitative biomarkers of thrombus composition in a single and fast scan. Combining MSTI with other clinical and molecular markers may enhance its predictive power, leading to a more comprehensive, personalized approach to DVT management. Finally, although this study focused on deep vein thrombosis in the leg, MSTI could potentially be applied to other thromboembolic conditions, such as pulmonary embolism and acute ischemic stroke, where thrombolysis is often considered. In these conditions, thrombus composition plays a critical role in determining treatment success, and the ability of MSTI to characterize thrombi in these vascular territories could help refine patient selection and improve outcomes. Further research into MSTI’s role in these settings is now warranted.

### Conclusions

MSTI is a novel, noncontrast MRI-based imaging technique that provides a noninvasive assessment of thrombus composition and accurately predicts thrombolytic success. By offering a more refined approach to patient selection for CDT, MSTI has the potential to significantly improve clinical outcomes in the management of DVT and reduce the incidence of PTS. The high sensitivity and specificity of MSTI, combined with its compatibility with standard clinical MRI systems, make it a valuable tool for guiding therapeutic decisions. As the first study to link multiparametric, contrast-free MRI to thrombus lytic behavior in vivo, this work supports the rationale for a prospective clinical trial of MSTI-based intervention planning. Further studies are now needed to validate MSTI in larger, more diverse patient cohorts and to explore its utility in other vascular conditions, such as pulmonary embolism and stroke, where thrombolysis is considered. Additionally, MSTI may play a key role in guiding the choice of emerging thrombus removal strategies, including mechanical and pharmacomechanical therapies.

## Article Information

### Author Contributions

Drs Saha, Phinikaridou, and Smith conceived and designed the study. Drs Phinikaridou, Saha, Smith, and Botnar secured funding. Drs Phinikaridou and Saha managed and supervised the project. Drs Silickas and Phinikaridou executed experiments, acquired data, and conducted data analysis. Drs Saha, Black, Karunanithy, Patel, and Modarai managed patient care in the project. Drs Phinikaridou, Saha, Karunanithy, and Black interpreted the data. Drs Phinikaridou and Andia performed statistical analyses. Drs Saha, Phinikaridou, Smith, and Andia drafted and critically revised the article. Dr Botnar provided expertise on MRI data acquisition. All authors read and approved the final article before publication.

### Sources of Funding

This work was partially funded by a British Heart Foundation Project grant (PG/15/89/31793 to Drs Phinikaridou, Saha, Smith, and Botnar), ANID a—Millennium Science Initiative Program—(ICN2021_004) to Drs Andia and Botnar, and the Fondo Nacional de Desarrollo Científico y Tecnológico (FONDECYT) 1180525 to Dr Andia.

### Disclosures

None.

### Supplemental Material

Supplemental Methods

Tables S1 and S2

Figures S1 -S3

Video S1

## Supplementary Material


