# Supplementary material for: Multiparametric Contrast-Free MRI Successfully Identifies Venous Thrombus Responsive to Lytic Therapy: From Mice to Humans
Source: Circ Cardiovasc Imaging. 2025 Nov 3;18(11):e018175. doi: 10.1161/CIRCIMAGING.125.018175 (PMC12622266; doi:10.1161/CIRCIMAGING.125.018175)
Supplement: Supplementary file 2 [file hci-18-e018175-s002.pdf]

SUPPLEMENTAL MATERIAL

Supplemental Methods

Animal Model

Venous thrombosis was induced in the inferior vena cava (IVC) of 12-week-old BALB/**c** mice in a surgical procedure that involves stenosis and endothelial disturbance as we have previously described[11,17,18]. Briefly, the infrarenal portion of the IVC was exposed through a midline laparotomy. A length of 5-0 polypropylene suture was placed alongside the IVC, and a 4-0 silk ligature (Ethicon Ltd) was passed around the IVC, incorporating the polypropylene suture just below the left renal vein. The ligature was tightened and tied, and the polypropylene was withdrawn, leaving a stenosis in the IVC causing a 90% reduction in blood flow. Endothelial damage to this portion of the IVC was then induced by the application of a neurosurgical vascular clip (Braun Medical) for 15s on 2 occasions, 30s apart.

DVT patients

All patients were discussed in a multidisciplinary team (MDM) meeting consisting of vascular surgeons, interventional radiologists, haematologists and specialist nurses. Indications, contraindications and the likehood of success of lysis were discussed before a recommendation was made to the patient by the clinical team. Prior to the start of thrombolysis, patients were approached about the participation in the study, if they met the inclusion criteria: adults aged 18 and over with a confirmed DVT suitable for lysis and able to provide informed consent. Exclusion criteria included those with SIRS (systemic inflammatory response syndrome), inflammatory diseases, malignancies, MR unsafe metal implants and phobia of small, enclosed spaces. Patient information sheets were provided to the patients prior to obtaining their written consent. Before enrolling patients into the main study, we conducted MSTI protocol testing in ten healthy volunteers. These scans were used to optimise sequence timing, imaging volume (renal veins to femoral vein), coil geometry, and patient positioning. This pilot step ensured MSTI was feasible in a clinical setting and reproducible across venous segments.

Magnetic Resonance Imaging (MRI) parameters

Murine imaging: Anesthesia was induced with 3-4% and maintained with 1-2% isoflurane during the MRI scan. Mice were scanned in supine position. Arterial and venous TOF angiography was performed as follows: arterial TOF with repetition time (TR)=40 ms, echo time (TE)=6.2 ms, flip angle=60°, field of view (FOV)=20×33×17 mm, acquired matrix=68×110, slice thickness=0.3 mm, resolution=0.3×0.3 mm, reconstructed resolution=0.1×0.1 mm, slices=50, averages=2, duration=7.5 minutes; and for venous TOF with TR=50 ms with all other parameters maintained resulting in a scan duration of 9mins. The maximum-intensity projection angiograms were used to visualise the abdominal aorta, the renal and iliac bifurcations and the venograms were used to visualise the inferior vena cava (IVC) and the region of flow obstruction corresponding to the thrombus. These images were used for planning of subsequent scans. T1-weighted spoiled 3D gradient-echo images were acquired ***(a)*** without and ***(b)*** with an on-resonance MT pre-pulse. The acquisition parameters were as follows: TR=115 ms, TE=16 ms, flip angle=18°, FOV=30×18×14mm, acquired matrix=148×89, slice thickness=0.4mm, acquired resolution=0.2×0.2mm, reconstructed resolution=0.1×0.1mm, slices=35, averages=1, and duration=6mins. The MT pre-pulse was a binomial block (1:2:1, 90°x 90°-x 90°-x 90°x) pulse with duration of 1.92ms and 1 repetition. 2D DW spin-echo images were acquired with TR=2.8 s, TE=105ms, flip angle=90°, diffusion echo time=333ms, FOV=18×30×12mm, acquired matrix=88×150, slice thickness=0.5mm, acquired resolution=0.2×0.2mm, reconstructed resolution=0.1×0.1mm, slices=24, averages=2, and duration=36mins. The apparent diffusion coefficient (ADC) was calculated from 4 b-values of 0, 333, 667, and 1000 mm^2/s. Diffusion gradients were applied parallel and perpendicular to the external magnetic field. T1 mapping was performed with a sequence that uses two non-selective inversion pulses with inversion times ranging from 20 to 2000ms, followed by 8 segmented readouts for eight individual images[11,18]. The two imaging trains result in a set of 16 images per slice with increasing inversion times. For T1 mapping, the acquisition parameters were TR=9.6ms, TE=4.9ms, TR between subsequent IR pulses=1000ms, flip angle=10°, FOV=36×22×10mm, acquired matrix=180×102, measured slice thickness=0.5mm, acquired resolution=0.2×0.2mm, reconstructed resolution=0.1×0.1mm, slices=20, and averages=1. The average velocity of blood was measured across the IVC using a phase-contrast sequence with the following

imaging parameters: spatial resolution=100×100µm, slice thickness=2mm, repetition time/echo time= 17.5/7.2ms, flip angle=30°, averages=6, and velocity encoding=15cm/s.

Human imaging: Patients were imaged in supine position for 40 minutes. Following a 3-dimensional (3D) gradient echo (GRE) scout scan, MR venography images were acquired to visualise the venous system in the segment of thrombus obstruction using a 3D balanced steady-state-free precession (bSSFP) and a 2D TOF scans. bSSFP images were acquired with FOV=220×299×200mm, matrix=112×148, in-plane resolution=2×2mm, slice thickness=2mm, repetition time/echo time (TR/TE)=4.2/2.1ms, flip angle=70°, T2-preparation=30ms, number of averages=1. 2D TOF images were acquired with FOV=220×299×200 mm, matrix=112×148, in-plane resolution=2×2 mm, slice thickness=4mm, TR/TE=7.3/3.5ms, flip angle=50° and number of averages=1. The maximum intensity projection images were used to plan the subsequent scans. T1-weighted spoiled 3D GRE images were acquired (a) without and (b) with an on-resonance magnetisation transfer (MT) pre-pulse[17]. The acquisition parameters were: FOV=220×299×200mm, matrix=112×148, in-plane resolution=2×2 mm, slice thickness=6 mm, TR/TE=69/2.2 ms, and flip angle=18°, number of averages=1. The MT pre-pulse was a binomial block (1:2:1, 90°x 90°-x 90°-x 90°x) pulse with duration of 1.92ms and 1 repetition. 2D diffusion weighted (DW) images were acquired with FOV=220×299×200mm, matrix=112×148, in-plane resolution=2×2 mm, slice thickness=10mm, TR/TE=1780/82ms, flip angle=90°, number of averages=2. The ADC was calculated from 4 b values of 0, 333, 667, and 1000 mm^2/s. Diffusion gradients were applied parallel and perpendicular to the external magnetic field. Finally, T1 mapping was performed using a 2D Modified Look-Locker Inversion Recovery (MOLLI) sequence and an acquisition scheme of 3-5[19,20] for T1 mapping, the acquisition parameters were FOV=220×299×200mm, matrix=112×148, in-plane resolution=2×2mm, slice thickness=6mm, TR/TE=3.3/1.6ms, TR between subsequent IR pulses=1000ms, flip angle=35°, number of averages=1.

Supplemental Figures and Figure Legends

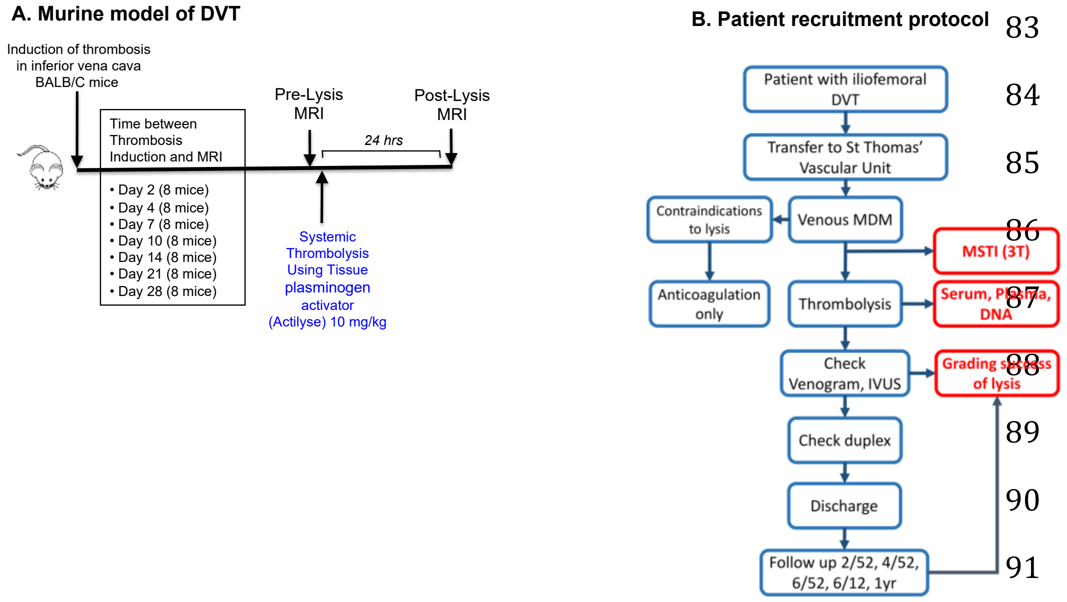

Figure S1: Study design. (A) MSTI protocol in the murine model of DVT. **(B)** Patient recruitment protocol.

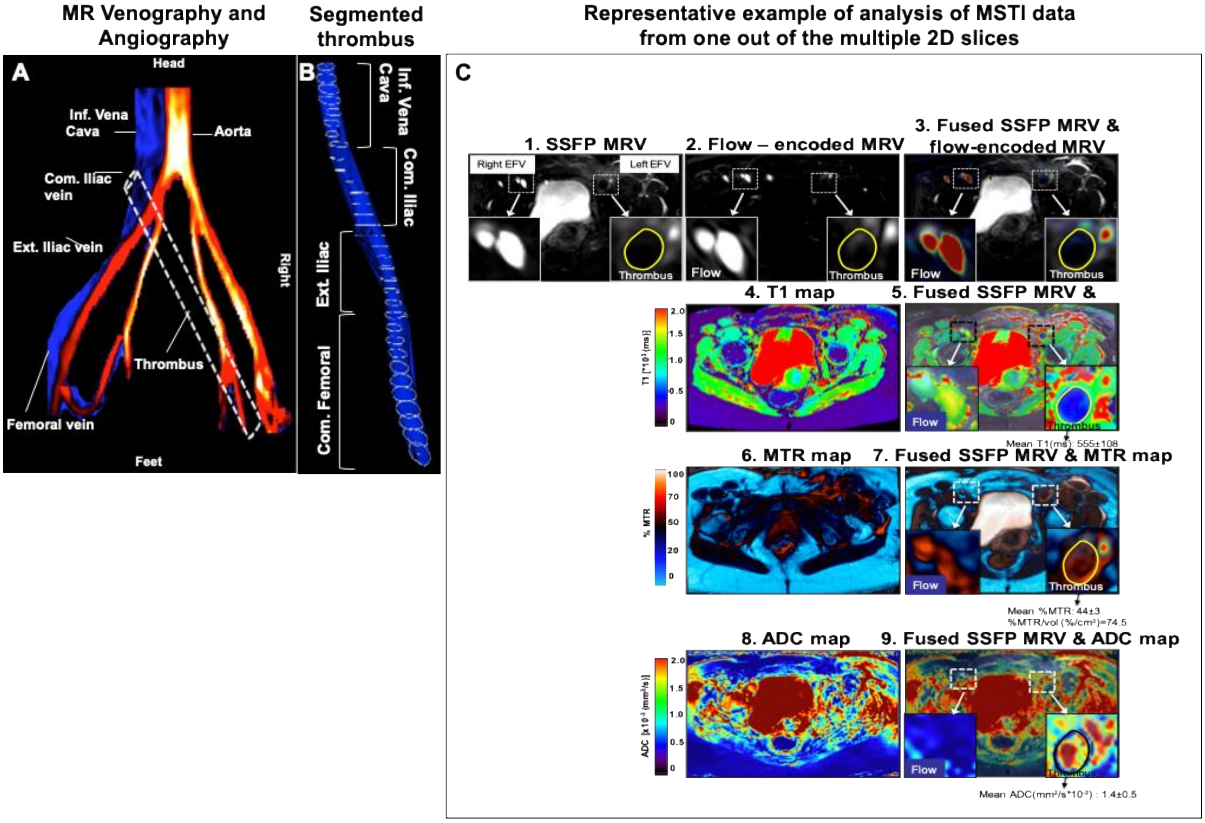

Figure S2: Segmentation of MR images acquired using the MSTI protocol to characterise thrombus in patients with DVT. (A) Recontraction of a stack of 2D images acquired with MR venography and angiography images. The images covering the vasculature extending from the inferior vena cava to the common femoral vein showing a filling defect corresponding to the thrombus. **(B)** Volumetric reconstruction of the thrombus segmented from continuous 2D slices. **(C)** Representative example of segmentation of MSTI images acquired at one of the multiple 2D images along the thrombus. In each patient, the MSTI-derived data were acquired from multiple, continuous 2D images covering the entire thrombus in the foot-head direction.

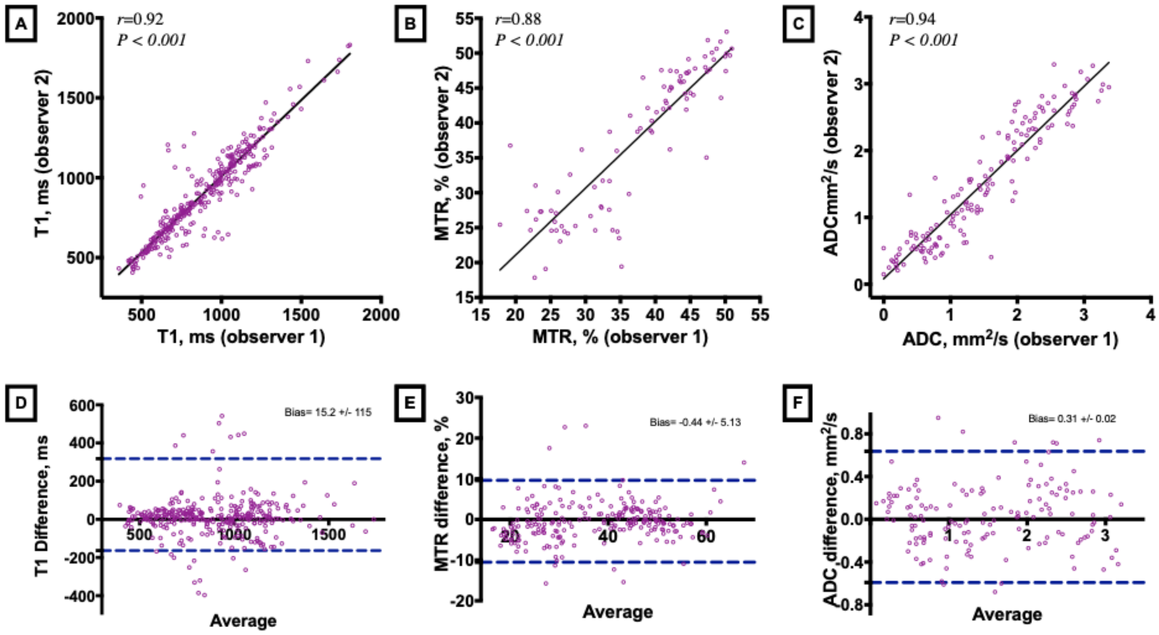

Figure S3: Inter-observer variability in analyzing MSTI images in patients with DVT.

Supplemental Tables

Table S1: Comparison of Mouse and Human MSTI Protocols

Step	**Mouse MSTI Protocol**	**Human MSTI Protocol**
MRI Scanner	3T MRI System	3T MRI System
Scout / Planning	3D GRE scout<br>2D TOF angiography/venography to identify IVC and thrombus filling defect	3D GRE scout<br>3D bSSFP venography and 2D TOF covering IVC to CFV, identifying thrombus filling defect
MSTI Sequences	3D MOLLI T1-mapping (two imaging trains followed by 8 segmented readouts for a total of 16 images/slice)<br><br>3D spoiled GRE magnetisation transfer (with/without MT pre-pulse)<br><br>2D multi-slice DWI (4 b-values: 0, 333, 667, 1000 mm²/s; parallel/perpendicular gradients)	2D multi-slice MOLLI T1-mapping (3-5 scheme)<br><br>3D spoiled GRE magnetisation transfer (with/without MT pre-pulse)<br><br>2D multi-slice DWI (4 b-values: 0, 333, 667, 1000 mm²/s; parallel/perpendicular gradients)
Outcome Assessment	Phase-contrast velocity mapping pre- and post-tPA to quantify IVC flow<br><br>Lysis defined as ≥50% increase in flow	Post-thrombolysis venography and IVUS<br><br>Lysis defined as <50% residual stenosis

Table S2: ROC analysis for MSTI measurements in patients with DVT.

	**T1**	**MTR**	**ADC**	**T1 + ADC**
**AUC**<br>**95% CI**	0.814<br>[0.721 – 0.907]	0.542<br>[0.431 – 0.634]	0.690<br>[0.581 – 0.798]	0.847<br>[0.764 – 0.930]
***P* value**	*< 0.0001*	*0.45*	*< 0.001*	*< 0.0001*

Video Legends

MSTI data acquisition protocol and images in patients with DVT.
